# Preparation of solvent-based fluorine–silicon modified acrylate and its application in specialty paper

**DOI:** 10.3389/fchem.2026.1943305

**Published:** 2026-10-30

**Authors:** Chenrong Yang, Shi Li, Yongheng Qiao, Xinbo Yu, Xinghui Yang, Meng Zhao, Pan Xie, Guiqiang Fei, Haihua Wang

**Affiliations:** 1 College of Chemistry and Chemistry Engineering, Shaanxi Key Laboratory of Chemistry Additives for Industry, Shaanxi University of Science & Technology, Xi’an, China; 2 Xi’an Aerospace Composites Research Institute, Xi’an, Shaanxi, China

**Keywords:** acrylate copolymer, fluorine–silicon modification, specialty paper, surface sizing agent, water resistance

## Abstract

Conventional starch and polyvinyl alcohol sizing agents rely on hydrogen bonding, but are susceptible to temperature and humidity, limiting their use in specialty papers such as banknotes. To address this, we synthesized a fluorine–silicon modified acrylate via radical copolymerization of hexafluorobutyl methacrylate (HFBMA) and silane coupling agents (KH570 and KH560) with common acrylate monomers, using ethanol as solvent. HFBMA lowers surface energy through C–F bond surface segregation, while the silane components form Si–O–Si networks and silanol groups that strengthen hydrogen bonding with cellulose fibers. Upon drying, the fluorinated and siloxane segments migrate to the paper surface and assemble into a hydrophobic barrier. Among three systems (F3, Si_570_-F3, and Si_560_-F3), the optimized Si_560_-F3 at 5% dosage increased tensile strength from 28.1 to 47.8 MPa (+70.1%), reduced water absorption from 29.1% to 5.5% (−81.1%), and raised water contact angle from 85° to 122°. These results confirm that this sizing agent effectively enhances both mechanical strength and water resistance for specialty paper.

## 1 Introduction

Specialty papers, including banknote paper, security paper, and energetic paper-based materials, require superior mechanical strength, dimensional stability, and water resistance to withstand demanding service conditions. Surface sizing is one of the most effective approaches for achieving these performance requirements (Jiang et al., 2025; Zhao et al., 2024a). Among commercially available sizing agents, starch-based products are the most widely used because of their low cost, abundant availability, and renewability, whereas poly (vinyl alcohol) (PVA) is a representative synthetic surface-sizing agent (Huang et al., 2023; Nizardo et al., 2024). Both materials reinforce paper primarily through hydrogen-bond interactions with cellulose fibers. However, the stability of these hydrogen bonds is highly susceptible to environmental temperature and humidity, resulting in limited long-term durability. In addition, native starch exhibits relatively poor sizing efficiency and high solution viscosity, which compromise processing stability and application performance. Although PVA provides superior film-forming capability, its poor biodegradability raises environmental concerns (Gengec et al., 2024; Zhang et al., 2025b). These inherent limitations restrict the ability of conventional sizing agents to satisfy the increasingly stringent durability and performance requirements of high-end specialty papers.

Acrylate polymers have attracted considerable attention because of their excellent film-forming ability, strong adhesion to cellulose fibers, and readily tunable properties through copolymerization (Shen et al., 2026). However, unmodified polyacrylates exhibit insufficient water resistance due to the presence of hydrophilic ester groups in their polymer chains. To address this issue, fluorine- or silicon-containing monomers have been introduced through copolymerization (Chen Y. et al., 2025). Fluorinated segments can migrate to the polymer surface to form a low-surface-energy layer, while silicon-containing components form a Si–O–Si crosslinked network, thereby synergistically improving hydrophobicity and mechanical properties (Zhou et al., 2024). Basu et al. (2012) prepared a superhydrophobic and oleophobic polydimethylsiloxane/silica nanocomposite coating using fluoroalkylsilane (FAS) as the topcoat. Lakshmi et al. (2012) fabricated a superhydrophobic sol–gel coating by incorporating fumed silica into a perfluoroalkyl methacrylate copolymer.

Despite these advances, current fluorosilicone composite sizing systems still have several limitations. Some modified materials primarily function through surface coating, resulting in the enrichment of hydrophobic components near the paper surface and limited modification of the internal fiber network. When the coating exhibits insufficient adhesion to the fiber matrix, its durability under prolonged friction or humid conditions may also be compromised. Therefore, achieving a balance between surface water resistance and internal structural modification remains a key challenge for these systems (Chen W. et al., 2025). The synergistic regulation of different functional components also requires further improvement. Fluorinated components can reduce surface energy and enhance hydrophobicity, while silicon-based components can improve the polymer network structure and interfacial properties. However, their incorporation does not simply result in an additive effect; the component ratio and crosslinking structure can significantly affect the final performance. Previous studies have shown that fluorosilicone synergistic modification can, to some extent, simultaneously improve hydrophobicity and mechanical properties, but further optimization is still needed to achieve a better balance between these properties (Minoofar et al., 2025). In terms of preparation, some fluorosilicone hybrid systems still rely on relatively complex processes, such as sol–gel methods, which impose certain requirements on reaction conditions and film formation. This may also increase the difficulty of integrating these systems with conventional paper sizing processes and continuous production. Therefore, simplifying the preparation process and improving process compatibility remain important practical considerations (Hadhri et al., 2025).

In this study, hexafluorobutyl methacrylate (HFBMA) was employed as the fluorinated monomer, while the silane coupling agents 3-Methacryloxypropyltrimethoxysilane (KH570) and 3-glycidyloxypropyl trimethoxysilane (KH560) were used as silicon-containing monomers, with ethanol serving as the solvent. A solvent-based fluorine–silicon modified acrylate resin was prepared to improve the penetration of the sizing agent into the paper substrate, thereby further enhancing its resistance to water and other polar substances. The incorporation of HFBMA, KH570, and KH560 improved the water resistance and thermal stability of the acrylate resin. Moreover, the mechanical properties and water resistance of the sized paper were significantly enhanced.

## 2 Materials and methods

### 2.1 Materials

Hexafluorobutyl methacrylate (HFBMA, industrial grade, Harbin Xuejia Fluorosilicon Co., Ltd., Harbin, China); acrylic acid (AA, CP grade, Tianjin Kermel Chemical Reagent Co., Ltd., Tianjin, China); butyl acrylate (BA, AR grade, Tianjin Oubokai Chemical Co., Ltd., Tianjin, China); methyl methacrylate (MMA, AR grade, Tianjin Oubokai Chemical Co., Ltd., Tianjin, China); 2-hydroxyethyl methacrylate (HEMA, AR grade, Tianjin Oubokai Chemical Co., Ltd., Tianjin, China); hydroxypropyl acrylate (HPA, AR grade, Tianjin Oubokai Chemical Co., Ltd., Tianjin, China); alkylphenol polyoxyethylene ether (OP-10, industrial grade, Xingtai Xinlanxing Technology Co., Ltd., Xingtai, China); allyloxy nonylphenol polyoxyethylene ether ammonium sulfate (DNS-86, industrial grade, Qingyuan Hanke Chemical Technology Co., Ltd., Qingyuan, China); acrylamide (AM, analytical grade, Tianjin Kermel Chemical Reagent Co., Ltd., Tianjin, China); *N,N′*-methylenebisacrylamide (MBA, analytical grade, Tianjin Kermel Chemical Reagent Co., Ltd., Tianjin, China); glycidyl methacrylate (GMA, analytical grade, Tianjin Kermel Chemical Reagent Co., Ltd., Tianjin, China). diethylene glycol dimethyl ether (DGDE, AR grade, Tianjin Kermel Chemical Reagent Co., Ltd., Tianjin, China); absolute ethanol (AR grade, Tianjin Kermel Chemical Reagent Co., Ltd., Tianjin, China); sodium bicarbonate (AR grade, Tianjin Tianli Chemical Reagent Co., Ltd., Tianjin, China); ammonium persulfate (APS, AR grade, Tianjin Tianli Chemical Reagent Co., Ltd., Tianjin, China); benzoyl peroxide (BPO, reagent grade, 99.0%, Shanghai Macklin Biochemical Co., Ltd., China); bleached kraft pulp (industrial grade, Dalian Yangrun Trading Co., Ltd., Dalian, China); and nitrocellulose (industrial grade, Northern Nitrocellulose Co., Ltd., China); 3-Methacryloxypropyltrimethoxysilane (KH570, 97%, Shandong Usolf Chemical Technology Co., Ltd., China); 3-glycidyloxypropyl trimethoxysilane (KH560, 97%, Shandong Usolf Chemical Technology Co., Ltd., China).

### 2.2 Preparation of waterborne acrylic resin binder G-AHBM

At room temperature, a defined amount of OP-10 and DNS-86 was dissolved in water in a three-necked flask. MMA, BA, GMA, HPA, and HEMA were mixed uniformly in a predetermined ratio, followed by the addition of AA to obtain the mixed oily monomers. One-tenth of the mixed oily monomers was introduced into the three-necked flask containing the dissolved emulsifiers and emulsified under high-speed stirring for 30 min. AM and MBA were dissolved in a quantitative amount of water in a predetermined ratio to prepare the mixed aqueous monomers. A quantitative amount of APS and NaHCO_3_ was dissolved in water to prepare the initiator aqueous solution. At 95 °C, one-third of the initiator aqueous solution was added to the emulsified emulsion to initiate the polymerization for 15 min. Subsequently, the remaining nine-tenths of the mixed oily monomers, the mixed aqueous monomers, and the remaining two-thirds of the initiator aqueous solution were simultaneously and slowly added dropwise into the stirred three-necked flask over a period of 150 min. After the dropwise addition, the reaction mixture was maintained at the same temperature for 0.5 h, then cooled to room temperature, and the pH of the emulsion was adjusted to neutral with ammonia water to obtain the G-AHBM binder (Liu, 2024). The composition formula is presented in Table 1.

**TABLE 1:** Synthetic formulation of water-based acrylic resin binder G-AHBM.

Component	OP-10	DNS-86	AM	MBA	BA	MMA	GMA	HPA	HEMA	AA	APS	NaHCO_3_
Mass Fraction (%)	0.6	2	4	2	41	41	5	4	2	2.5	0.5	0.5

### 2.3 Preparation of fluorinated acrylate copolymers

Fluorinated acrylate copolymers were synthesized using MMA, BA, HPA, and HFBMA as the main monomers. BPO (1.2–1.8 wt% of the total monomer mass) was used as the initiator. DGDE and absolute ethanol were used as the solvent and dispersing agent, respectively. The initiator was added to the mixed monomers, and the mixture was sonicated for 5 min to ensure homogeneous mixing and complete dissolution of the initiator. A three-necked flask containing DGDE was placed in an electrically heated mantle equipped with a mechanical stirrer and heated to 110 °C–120 °C. The monomer mixture containing the dissolved initiator was transferred to a constant-pressure dropping funnel and added dropwise to the flask over 2.5–3 h. After the addition was completed, the reaction mixture was maintained at 120 °C for 1 h. Subsequently, the temperature was reduced to 50 °C–60 °C, followed by the addition of absolute ethanol, and the mixture was stirred thoroughly for 15–20 min. The synthesized fluorinated acrylate copolymers were designated as Fn, where n represents the mass fraction of HFBMA relative to the total mass of the other monomers. The HFBMA contents were 0%, 10%, 20%, 30%, and 40%, and the corresponding copolymers were designated as F0, F1, F2, F3, and F4, respectively. The schematic illustration of the synthesis is shown in Figure 1. The composition formula is presented in Table 2.

**FIGURE 1:**
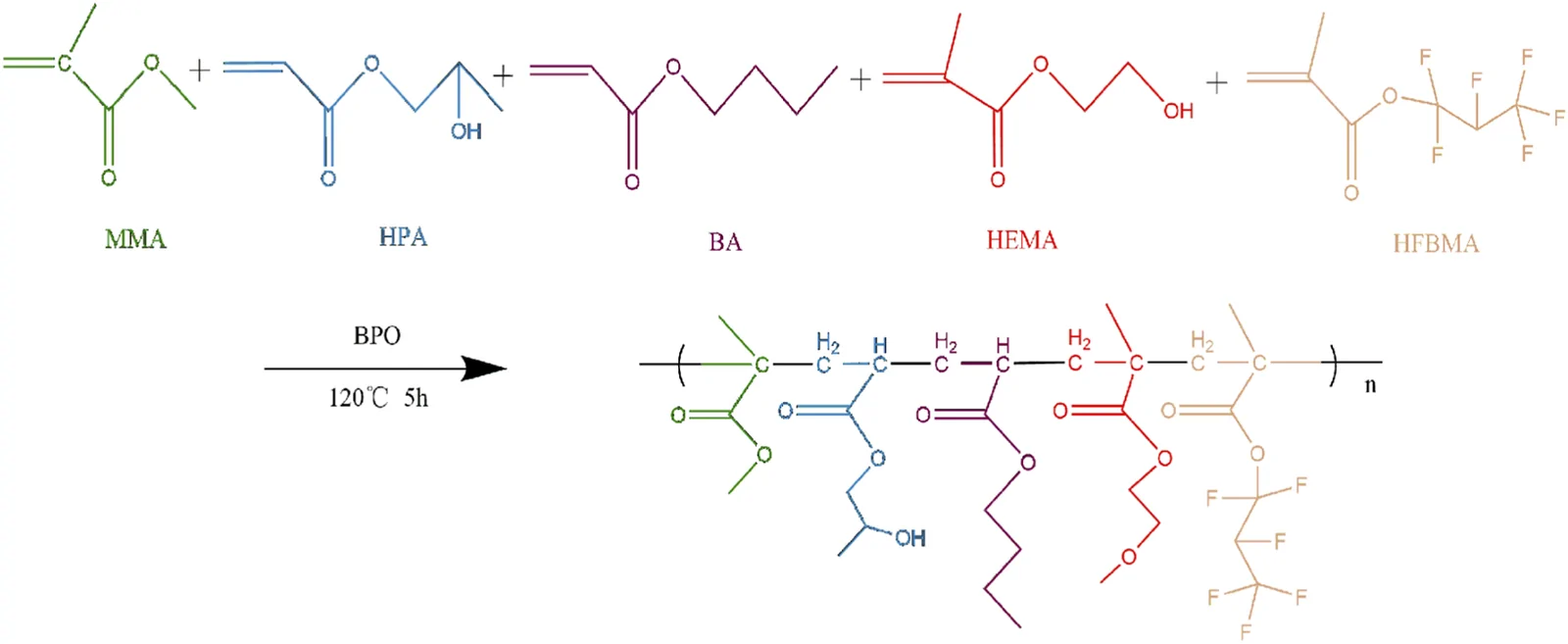
Polymerization mechanism of fluorine-modified acrylic resin.

**TABLE 2:** Synthetic formulation of fluorinated acrylate copolymers.

Component	HFMBA (g)	MMA (g)	BA (g)	HPA (g)	HEMA (g)
F0	0	8	12	5	5
F1	3.33	8	12	5	5
F2	7.50	8	12	5	5
F3	12.86	8	12	5	5
F4	20	8	12	5	5

### 2.4 Preparation of fluorine–silicon Co-Modified acrylate resins

Two different organosilane coupling agents were used to modify the fluorinated acrylate sizing agents. Method I. A predetermined amount of KH570 was introduced into the optimized formulation described in Section 2.3, and a fluorine–silicon co-modified acrylate resin was synthesized via free-radical polymerization. The obtained resin was designated as nSi_570_-F3. Method II. The fluorinated acrylate resin was modified through the reaction of the epoxy groups and highly reactive silanol groups of KH560 with the hydroxyl groups in the polymer chains. Based on the optimized polymerization conditions described in Section 2.3, an appropriate amount of KH560 was added after the complete dropwise addition of the monomer mixture. The reaction mixture was maintained at 120 °C for 1 h, followed by the addition of absolute ethanol to adjust the resin to the desired solid content. The obtained resin was designated as nSi_560_-F3, where n represents the mass fraction of the organosilane coupling agent relative to the total monomer mass. The schematic illustration of the synthesis is shown in Figure 2. The composition formula is presented in Tables 3, 4.

**FIGURE 2:**
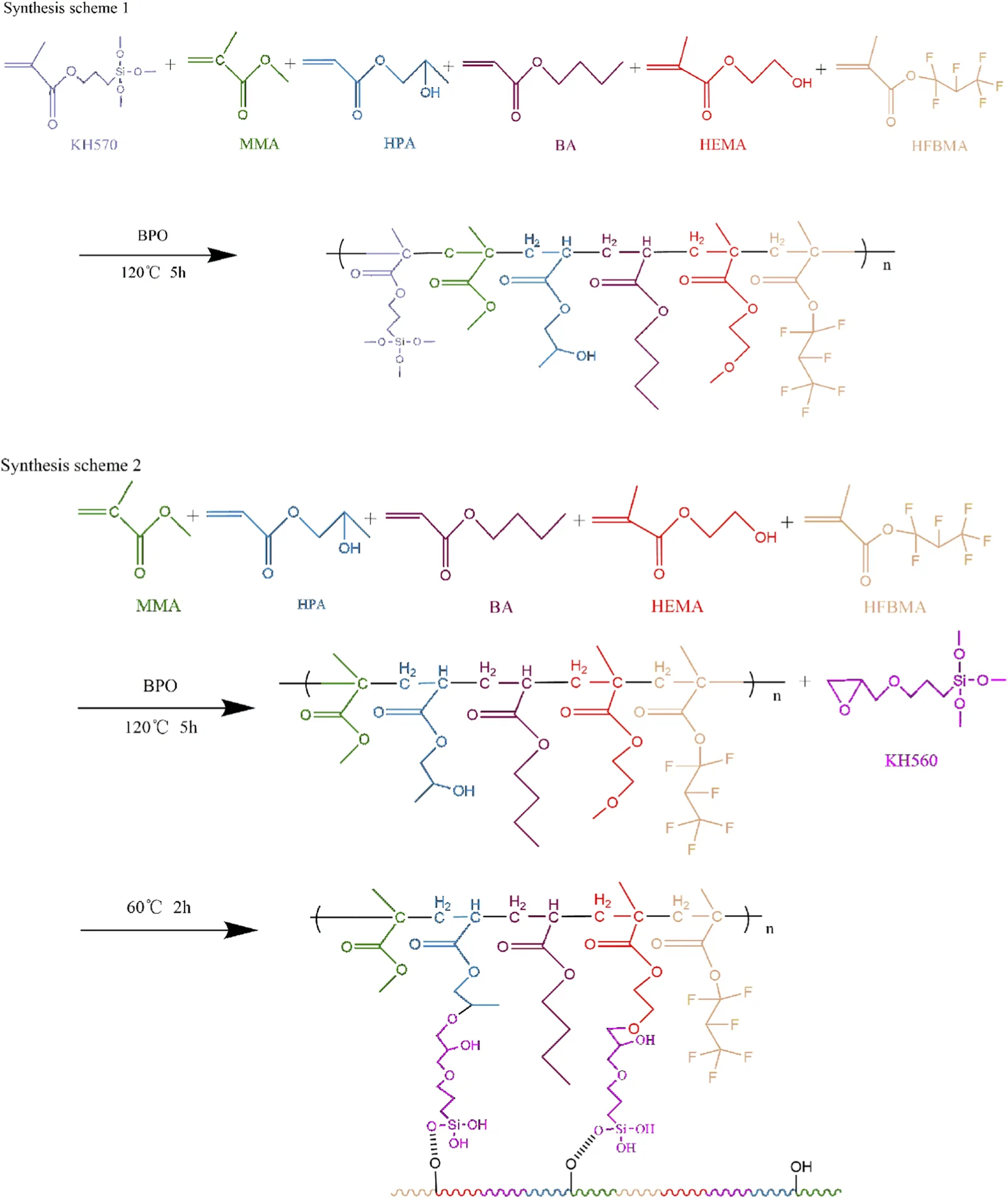
Polymerization mechanism of fluoro-silicon modified acrylic resin.

**TABLE 3:** Synthetic formulation of nSi_570_-F3.

Component	KH570 (%)	HFMBA (g)	MMA (g)	BA (g)	HPA (g)	HEMA (g)
0.5%Si_570_-F3	0.5%	12.86	8	12	5	5
1% Si_570_-F3	1%	12.86	8	12	5	5
1.5%Si_570_-F3	1.5%	12.86	8	12	5	5

**TABLE 4:** Synthetic formulation of nSi_560_-F3.

Component	KH560 (%)	HFMBA (g)	MMA (g)	BA (g)	HPA (g)	HEMA (g)
1%Si_560_-F3	1%	12.86	8	12	5	5
3%Si_560_-F3	3%	12.86	8	12	5	5
5%Si_560_-F3	5%	12.86	8	12	5	5
7%Si_560_-F3	7%	12.86	8	12	5	5

### 2.5 Preparation of specialty paper

First, a predetermined amount of pulp was accurately weighed according to the formulation and dispersed in approximately 1 L of water. The pulp suspension was then transferred to a fiber disintegrator and disintegrated at a stirring speed of 6000 rpm. After disintegration, a predetermined amount of G-AHBM binder was gradually added under continuous stirring, and the mixture was stirred until homogeneous. The resulting slurry was then transferred to a sheet former, and water was added to a total volume of 4 L. After thorough mixing, the drainage valve was opened to remove the water and form the wet paper sheet. The drainage valve was then closed, and the cylinder was opened. The wet sheet was covered with a filter cloth and gently rolled with a couch roll. Subsequently, the wire screen and wet sheet were removed. The sheet was inverted onto the workbench, couched, and covered successively with a filter cloth and a felt. The felt and wet paper sheet were then pressed together in a conventional press for 3 min. Finally, the paper sheet, together with the filter cloth, was hot-pressed in a flat-plate vulcanizing press at 95 °C under a pressure of 100 kg cm^-2^ for 6 min, followed by drying in a vacuum oven at 50 °C.

### 2.6 Surface sizing process

The paper samples were surface-sized using a spray-coating method. First, the sizing agent was prepared with a solid content of 20% and loaded into a precision spraying device. The paper samples were placed horizontally on the operating platform of a constant-temperature and constant-humidity fume hood under ambient conditions, followed by directional spraying. During spraying, the spray nozzle was maintained at a constant distance of 3.0 ± 0.5 cm from the paper surface and moved at a uniform speed in a reciprocating manner to ensure uniform distribution of the atomized droplets over the sample surface. The front and back sides of each paper sample were alternately spray-coated using a multiple-pass spraying procedure, with the total coating amount controlled below 2 wt%, based on the oven-dry pulp mass. After sizing, the samples were leveled and transferred to a forced-air drying oven for drying at 50 °C for 4 h. The dried samples were then conditioned under standard laboratory conditions (23 °C ± 1 °C) for 24 h. Finally, the conditioned samples were cut into standard dumbbell-shaped specimens using a computer-controlled film-cutting machine for subsequent mechanical property characterization.

### 2.7 Material characterization and performance tests

Fourier transform infrared (FTIR) spectroscopy: The chemical structures of the prepared sizing agents were characterized using a VECTOR-22 Fourier transform infrared spectrometer (Bruker, Germany). The spectra were recorded over the range of 4000–400 cm^-1^ with a resolution of 4 cm^-1^. X-ray photoelectron spectroscopy (XPS): The surface elemental composition and chemical bonding states of the samples were analyzed using an AXIS SUPRA X-ray photoelectron spectrometer (Kratos Analytical Ltd., United Kingdom). Scanning electron microscopy (SEM):The morphologies of the samples were observed using a MIRA field-emission scanning electron microscope (TESCAN, Czech Republic). Thermogravimetric analysis (TGA): Thermal stability was evaluated using a TGA Q500 thermogravimetric analyzer (TA Instruments, United States of America). The measurements were carried out under a nitrogen atmosphere from room temperature to 700 °C at a heating rate of 10 °C min^-1^. Gel permeation chromatography (GPC): The number-average molecular weight (Mn) and polydispersity index (PDI = M_w_/M_n_) were determined using a Waters 1525 gel permeation chromatography system (Waters, United States of America). The samples were dissolved in *N,N*-dimethylformamide (DMF) prior to analysis. Rheological measurements: The rheological behavior of the sizing agents was measured using an AR 2000ex rheometer at 50 °C over a shear-rate range of 3–400 s^-1^. Water absorption: The samples were cut into 2 cm × 2 cm squares and immersed in deionized water at room temperature for 24 h. After immersion, the samples were removed from the water, and the surface moisture was immediately removed with filter paper. The masses before and after immersion were recorded as m_1_ and m_2_, respectively. The water absorption of the paper was calculated using the following equation:

$$\text{Water absorption }\left(\%\right)=\frac{m_{2}-m_{1}}{m_{1}}*100\%$$

Water contact angle (WCA): Static water contact angles were measured using a CA500 contact angle goniometer (Shanghai Aifei Precision Instrument Co., Ltd., China). The samples were cut into 10 mm × 20 mm specimens. A water droplet with a volume of 4 μL was deposited on the sample surface. Each sample was measured three times, and the average value was reported. Tensile properties: Tensile tests were performed using an AI-7000-NGD servo universal testing machine (Gaotewell (Dongguan) Co., Ltd., China). The samples were cut into dumbbell-shaped specimens with dimensions of 45 mm × 75 mm and tested at a crosshead speed of 50 mm min^-1^. Three measurements were conducted for each sample, and the average value was reported. Dry and wet tensile specimens were randomly cut from circular handsheets. The handsheets were prepared via the Rapid-Köthen method (ISO 5269-2), which produces nearly isotropic paper with randomly distributed fibres without inherent machine direction (MD) and cross direction (CD). Quantitative mechanical data were obtained from independent replicate measurements and are expressed as mean ± standard deviation (SD). The apparent Young’s modulus was calculated from the slope of the initial approximately linear region of the stress–strain curve.

## 3 Results and discussion

### 3.1 Effects of key factors on the properties of fluorinated polyacrylate sizing Agents (Fn)

#### 3.1.1 Structural characterization of Fn sizing agents

The absorption band at 1734 cm^-1^ is attributed to the bending vibration of the C=O group. The bands at 1454 and 1388 cm^-1^ correspond to the stretching vibrations of CH_2_ groups. A characteristic absorption peak of the C–F bond is observed at 690 cm^-1^. In addition, the bands at 1284 and 1214 cm^-1^ are assigned to the symmetric and asymmetric stretching vibrations of the C–F bond, respectively. Although the C–F bond also exhibits an absorption band at 1164 cm^-1^, this signal is overlapped by the absorption band arising from the C–O stretching vibration. The presence of the C–F absorption bands confirms the successful incorporation of fluoroalkyl groups into the acrylate sizing agents (Tan et al., 2024). Furthermore, no characteristic absorption peak corresponding to the carbon–carbon double bond (C=C) is observed in Figure 3, indicating that all monomers were successfully polymerized (Zhang and Chen, 2005).

**FIGURE 3:**
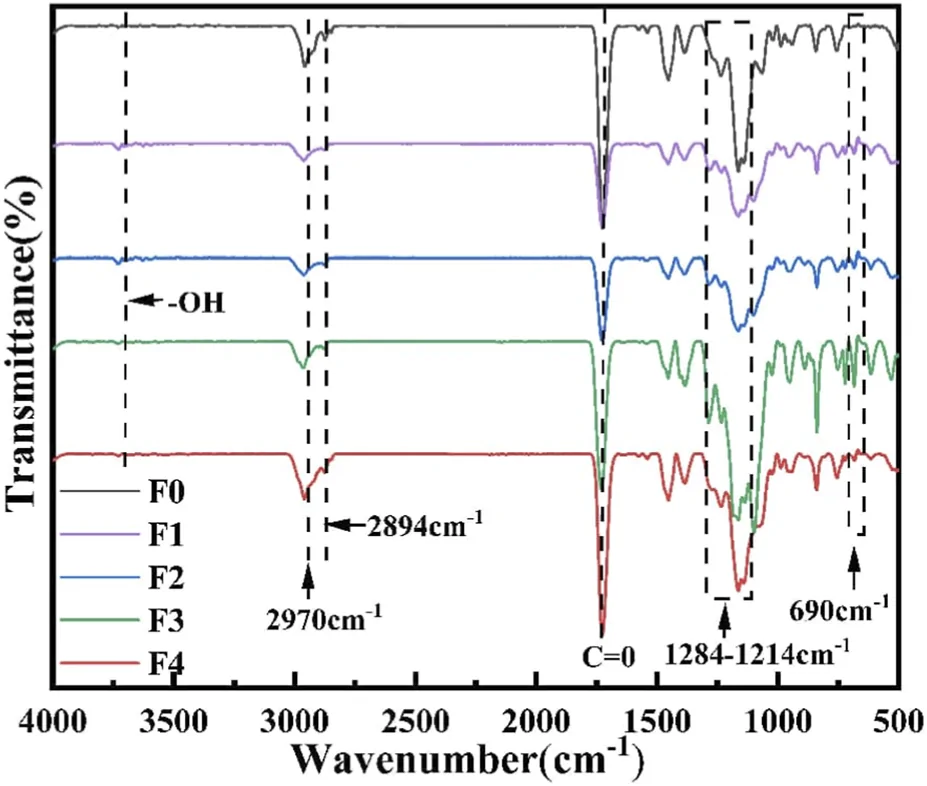
FT-IR spectra of sizing agent Fn.

#### 3.1.2 Effect of HFBMA content on the properties of Fn sizing agents

As shown in Figure 4a, the viscosity of the sizing agent decreased with increasing shear rate, indicating typical shear-thinning behavior. Flow occurred only when the applied shear stress exceeded the yield stress (Zhang et al., 2025c). This behavior can be attributed to the highly cohesive internal structure of the fluid, intermolecular hydrogen bonding, partial crosslinking, and the highly branched side chains in the absence of an external force (Zheng et al., 2025). As the content of HFBMA increased, the viscosity of the sizing agent first decreased and then increased. The lowest viscosity was obtained at an HFBMA content of 30 wt%.

**FIGURE 4:**
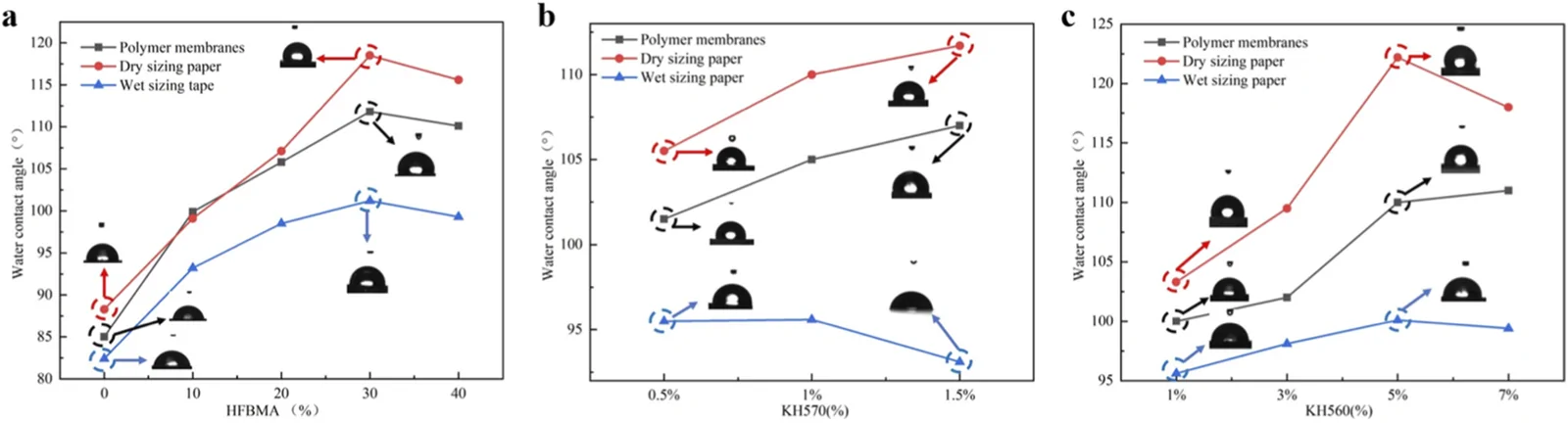
**(a)** Effect of HFBMA on rheological properties of sizing agent; **(b)** Effect of HFBMA on water absorption of sizing agent; **(c)** Thermogravimetric curve of Fn adhesive film.

For specialty paper applications, sizing agents are required to maintain stable performance under relatively harsh conditions, such as high temperature and high humidity (Zhang et al., 2025d). Therefore, the water absorption of the polymer films was evaluated by immersing the samples in water at room temperature for 24 h. As shown in Figure 4b, the water absorption of the unmodified film (F0) was significantly higher than that of the fluorinated films. Most of the water uptake occurred within the first 7 h, with the F0 film exhibiting the highest water absorption of 17.6%. As the HFBMA content increased, the water absorption gradually decreased. This behavior can be attributed to the extremely low surface energy of the fluorine atoms in HFBMA, while the fluoroalkyl segments incorporated into the polymer chains effectively hindered the penetration of water molecules (Zhao et al., 2024b). When the HFBMA content reached 30 wt%, the water absorption stabilized at approximately 2.6%. Although a further increase in the HFBMA content led to a slight reduction in water absorption, the improvement was marginal. Considering the cost of the sizing agent, the HFBMA content was fixed at 30 wt% in the subsequent experiments.

To investigate the effect of HFBMA on the thermal stability of the acrylate resin, thermogravimetric analysis (TGA) was performed, and the results are presented in Figure 4c. The incorporation of HFBMA shifted the TGA curves toward higher temperatures, indicating an improvement in the thermal stability of the acrylate resin. This enhancement is attributed to the high bond energy of the C–F bonds in the fluoroalkyl groups, which protect the polymer backbone during thermal degradation (Chen et al., 2023; Çınar and Karataş, 2023). Before 296 °C, the unmodified acrylate resin exhibited only a slight mass loss, mainly due to the evaporation of moisture from the polymer film. A pronounced weight loss was observed between 296 °C and 400 °C, which was mainly attributed to the thermal decomposition of the soft segments in the polymer chains. In the temperature range of 400 °C–460 °C, the decomposition was primarily associated with the cleavage of C–C, C–O, and C–H bonds in the polymer chains. Thermal decomposition was essentially completed above 460 °C.

#### 3.1.3 Effect of MMA:BA Ratio on the properties of Fn sizing agents

The molecular weight distribution and polydispersity of the acrylate sizing agents were characterized by gel permeation chromatography (GPC). As shown in Table 5, the glass transition temperature of polymer films can be effectively regulated by adjusting the ratio of soft to hard monomers (Lv et al., 2024; Ramasinghe et al., 2025). Accordingly, a series of F3 sizing agents with different soft-to-hard monomer ratios were prepared, and their molecular weights and water contact angles were evaluated. The fluorinated acrylate resins exhibited number-average molecular weights (M_n_) ranging from 6382 to 17,633 Da, with polydispersity indices (PDI) between 2.21 and 3.76, indicating a relatively uniform molecular weight distribution. The results also showed that varying the soft-to-hard monomer ratio had little influence on the water contact angle. However, repeated experiments demonstrated that a soft-to-hard monomer ratio of 1:1 produced a polymer film with suitable flexibility and rigidity, making it more appropriate for paper surface sizing.

**TABLE 5:** Effect of hard and soft monomer ratio on sizing agent performance.

MMA:BA	Mw (g/mol)	Mn (g/mol)	PDI	WCA (°)
3:0	23,704	10,680	2.21	82.1
3:1	39,811	17,633	2.25	83.4
2:1	21,444	6382	3.36	85.2
1:1	25,233	6701	3.76	85.3
1:2	24,408	11,530	2.11	79.5

### 3.2 Effect of KH560 on the properties of F3 sizing agent

#### 3.2.1 Structural characterization of Si_560_-F3

Figure 5 shows the FTIR spectra of F3 and Si_560_-F3 samples with different KH560 contents. The absorption bands at approximately 2970 and 2894 cm^-1^ are assigned to the stretching vibrations of aliphatic -CH_3_ and -CH_2_ groups, while the strong band near 1730 cm^-1^ corresponds to the ester C=O stretching vibration of the acrylate backbone. The bands at 1284 and 1214 cm^-1^ are attributed to the characteristic stretching vibrations of C-F bonds. These bands are observed in both the F3 control and all Si_560_-F3 samples, indicating that the fluorinated segments remain present after KH560 modification. Compared with F3, the Si_560_-F3 samples exhibit additional changes in the fingerprint region. The absorption near 912 cm^-1^ is associated with the epoxy structure of KH560, while the bands around 842 and 750 cm^-1^ are related to silicon-containing structures introduced by the silane coupling agent (Kogje et al., 2025). These spectral features support the successful incorporation of KH560 into the fluorinated acrylate system (Zhang et al., 2025a). It is worth noting that the apparent intensities of the C–F-related bands do not vary monotonically with increasing KH560 content. In particular, the bands at 1284 and approximately 1214 cm^-1^ are more intense for the 3% Si_560_-F3 sample than for the 5% sample. Since the HFBMA content was kept constant throughout the Si_560_-F3 series and only the KH560 content was varied, this difference should not be interpreted as indicating a higher fluorine content in the 3% sample. The absolute intensity of FTIR absorption bands can be affected by sample thickness, sample–crystal contact, baseline correction, and overlapping absorptions in the fingerprint region. In particular, the 1200–1300 cm^-1^ region contains overlapping contributions from C-F stretching and ester-related vibrations.

**FIGURE 5:**
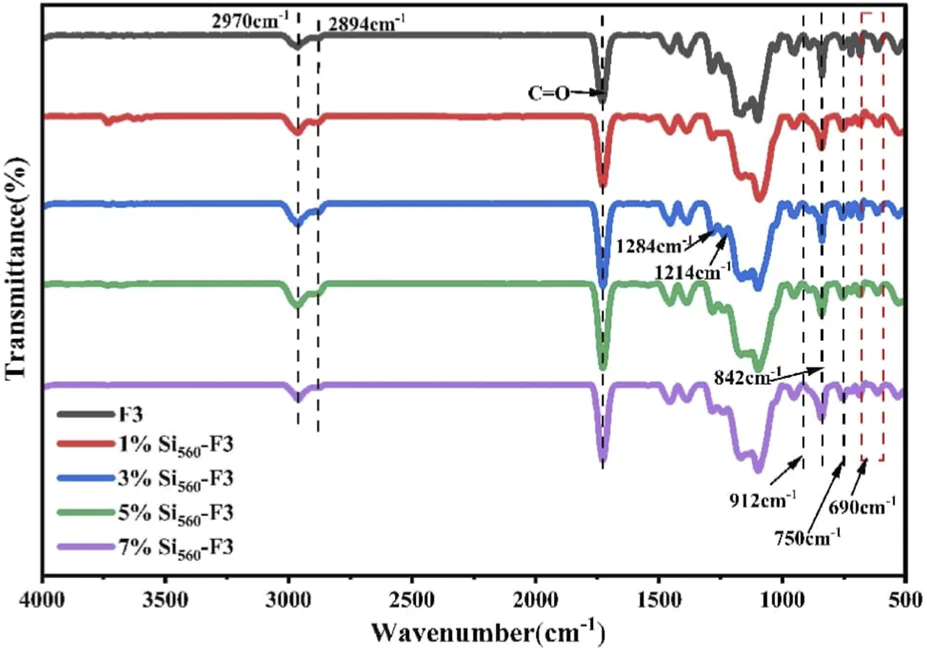
FTIR spectra of F3 and Si_560_-F3 sizing agents with different KH560 contents.

#### 3.2.2 Effect of KH560 on the rheological properties of F3 sizing agent

As shown in Figure 6, the viscosity of the sizing agent initially increased and then decreased with increasing KH560 content. As the KH560 content increased from 1 wt% to 7 wt%, the overall variation in viscosity was relatively small, with the maximum viscosity observed at a KH560 content of 5 wt%. The epoxy groups in KH560 can interact with the hydroxyl groups in the polymer chains, leading to the formation of hydrogen bonds (Ma et al., 2024b). However, hydrogen bonds are much weaker than covalent bonds. Therefore, as the shear rate increased, these weak intermolecular interactions were gradually disrupted, resulting in a decrease in viscosity (Chen et al., 2024).

**FIGURE 6:**
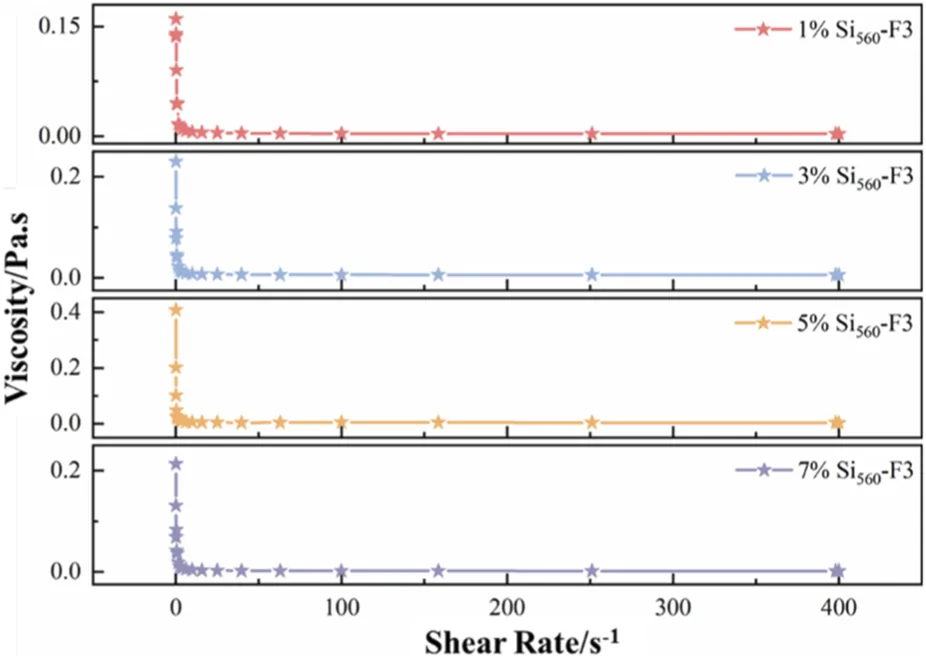
Rheological curves of Si_560_-F3 sizing agent.

#### 3.2.3 Effect of KH560 content on the molecular weight of F3 sizing agent

One of the characteristics of free-radical polymerization is the rapid propagation of polymer chains, resulting in a substantial increase in molecular weight. The molecular weight distribution plays an important role in determining the physical and mechanical properties of polymers, such as impact strength and glass transition temperature (Bamane et al., 2024). As shown in Table 6, the unmodified fluorinated acrylate sizing agent exhibited a number-average molecular weight (M_n_) of approximately 6382 Da. After the incorporation of KH560, the Mn increased to 6701–13200 Da, while the polydispersity index (PDI) ranged from 3.0 to 4.0, indicating a broad molecular weight distribution and relatively poor molecular weight uniformity. The relatively high M_n_ and PDI values may be attributed to chain branching or crosslinking reactions, which resulted in the formation of polymers with exceptionally high molecular weights (Morgen and Mecking, 2024). When the polymerization was carried out at 120 °C–130 °C, pronounced crosslinking was observed, and the viscosity of the reaction system reached the practical limit for mechanical stirring.

**TABLE 6:** Molecular weight distribution of Si_560_-F3.

Sample	M_w_ (g/mol)	M_n_ (g/mol)	PDI
1% Si560-F3	34,051	8860	3.80
3% Si560-F3	49,380	13,200	3.70
5% Si560-F3	25,233	6701	3.70
7% Si560-F3	42,480	11,491	3.60

#### 3.2.4 Effect of KH560 content on the thermal stability of F3 sizing agent

As shown in Figure 7, the TGA curves of the fluorinated acrylate resins shifted slightly toward higher temperatures with increasing KH560 content, although the overall change was limited. All samples exhibited similar thermal degradation profiles. The major weight loss occurred between 310 °C and 400 °C, corresponding to the thermal decomposition of the soft segments in the polymer chains. In addition, the onset decomposition temperature gradually increased with increasing KH560 content. This improvement is mainly attributed to the Si–O–Si network formed by KH560, which increased the crosslinking density of the polymer. The resulting crosslinked structure restricted the mobility of the polymer chains at elevated temperatures, thereby retarding thermal degradation and improving the thermal stability of the polymer films (Zhang et al., 2024).

**FIGURE 7:**
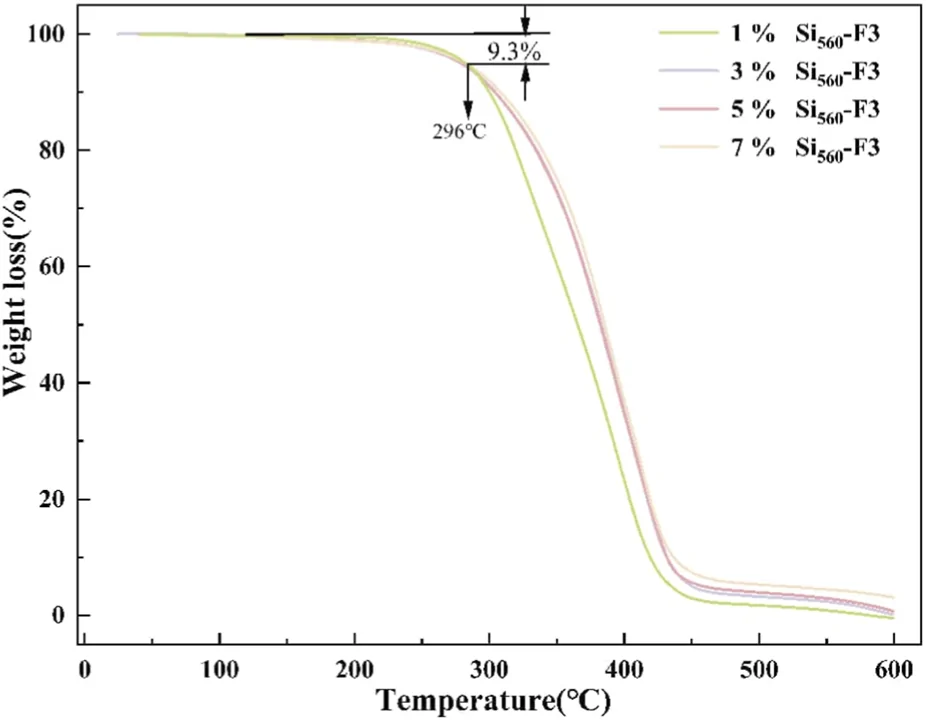
Thermogravimetric curve of Si_560_-F3 adhesive film.

#### 3.2.5 Effect of KH560 content on the water absorption of F3 sizing agent

Figure 8 shows the 24 h water absorption of the KH560-modified polymer films. The water absorption decreased initially with increasing KH560 content and reached a minimum of approximately 1.3% at 5 wt%, followed by a slight increase at higher KH560 contents. The improved water resistance is attributed to the denser network formed during crosslinking after epoxy ring opening. Meanwhile, the increased crosslinking density favored the enrichment of C–F bonds and Si–O–Si segments at the film surface, which further inhibited water penetration (Zuo and Zhou, 2024).

**FIGURE 8:**
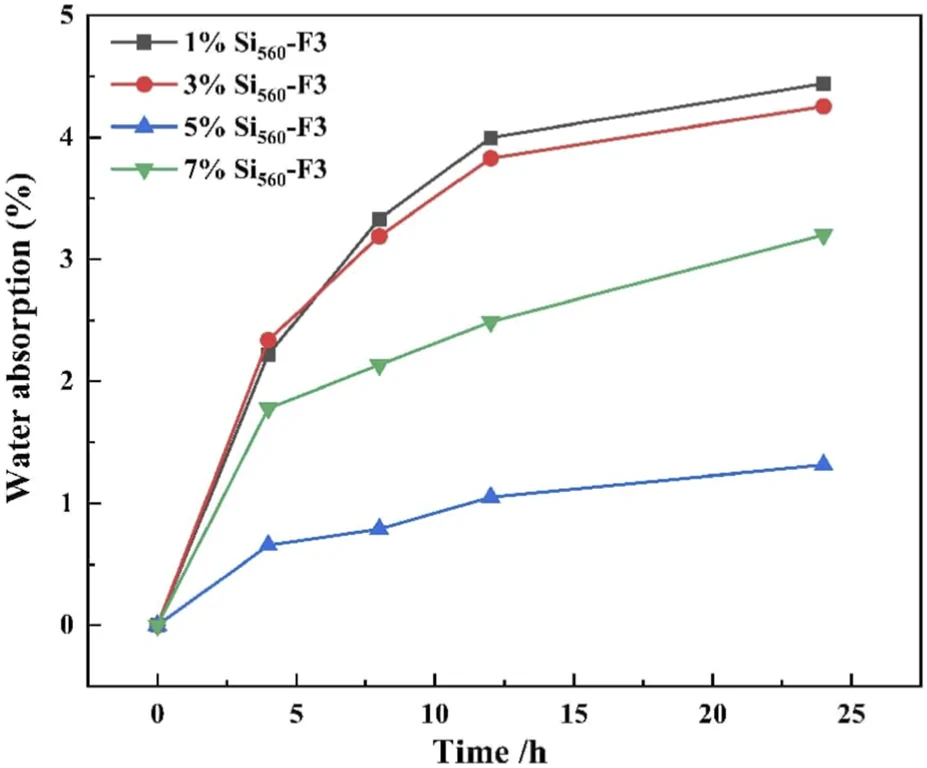
Effect of KH560 on the water absorption of adhesive film.

### 3.3 Effect of KH570 on the properties of F3 sizing agent

#### 3.3.1 Structural characterization of Si_570_-F3

Figure 9 shows the FTIR spectrum of the KH570-modified F3 sizing agent. The broad band at approximately 3344 cm^-1^ is assigned to the stretching vibration of hydroxyl groups. Absorption bands at 2970 and 2894 cm^-1^ arise from the stretching vibrations of methyl and methylene groups, and the strong band at 1730 cm^-1^ corresponds to the ester carbonyl (C=O) group. Compared with F3, the Si570-F3 samples showed more pronounced changes in the fingerprint region. The absorption bands at approximately 842, 811, 750, and 690 cm^-1^ are associated with silicon-containing structures introduced by KH570 and became more evident after silane modification. These spectral changes support the successful incorporation of KH570 into the fluorinated acrylate system.

**FIGURE 9:**
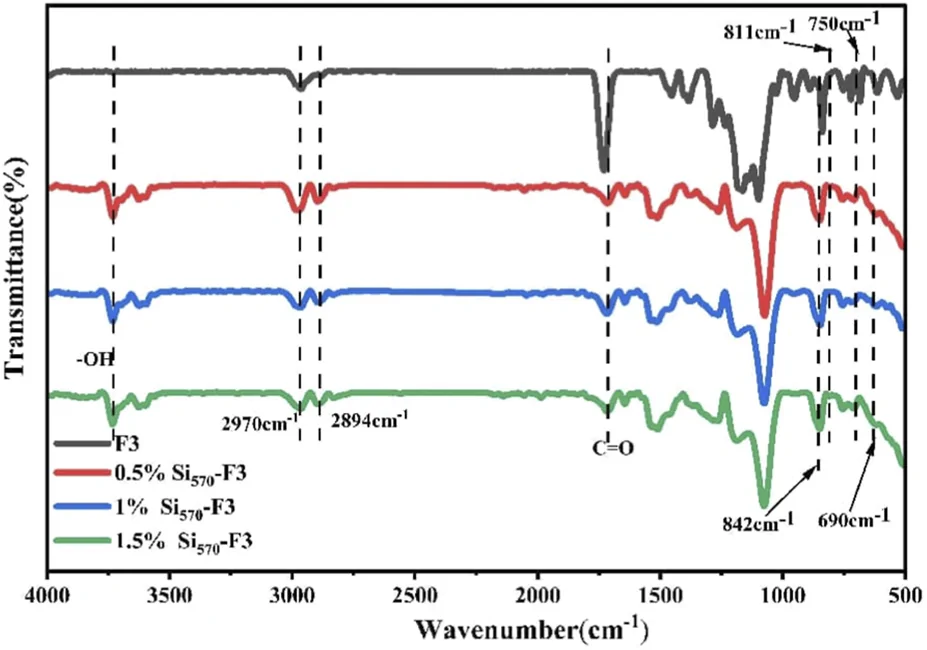
Infrared spectra of Si_570_-F3 sizing agent.

#### 3.3.2 Effect of KH570 on the rheological properties of F3 sizing agent

As the shear rate increased, the viscosity of the sizing agent decreased, exhibiting typical pseudoplastic behavior (Wang et al., 2024b). As shown in Figure 10, the viscosity increased with increasing KH570 content. This behavior is attributed to the participation of KH570 in the polymerization of the main chain through its carbon–carbon double bond, which increased the molecular weight of the polymer. Since viscosity generally increases with molecular weight, a corresponding increase in viscosity was observed. When the KH570 content reached 1.5 wt%, the viscosity increased sharply, which was likely due to the higher degree of crosslinking, resulting in the formation of high-molecular-weight polymers and chain entanglement. Further increasing the KH570 content reduced the stability of the system and led to gelation.

**FIGURE 10:**
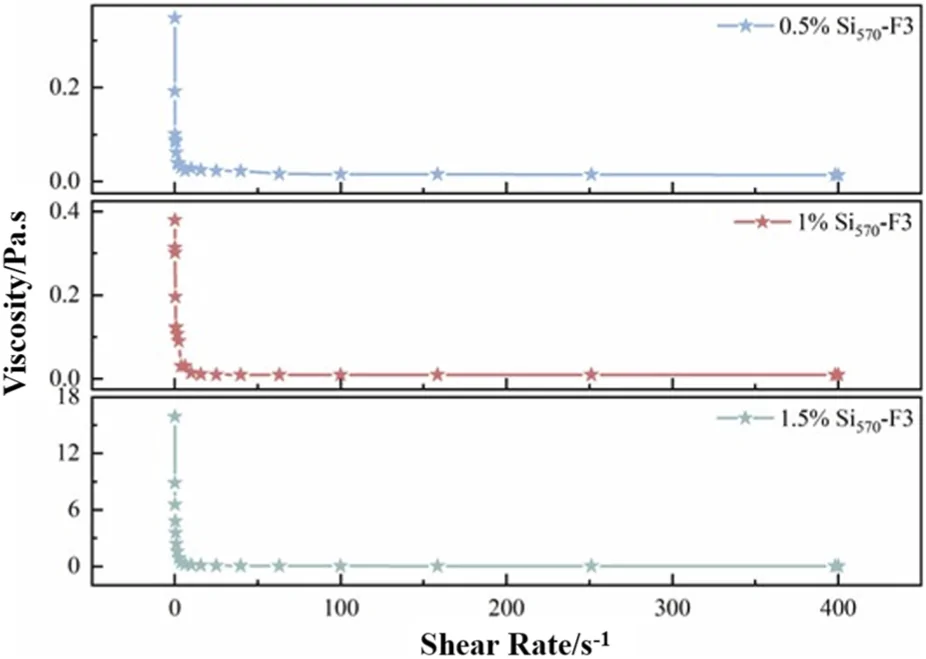
Rheological curve of Si_570_-F3 sizing agent.

#### 3.3.3 Effect of KH570 content on the molecular weight of F3 sizing agent

As shown in Table 7, the F3 sizing agent exhibited a number-average molecular weight (Mn) of approximately 6382 Da with a polydispersity index (PDI) of 3.36. After the incorporation of KH570, the Mn increased to 14,302–25033 Da, accompanied by a markedly broader molecular weight distribution, with the PDI reaching as high as 21.56. These results suggest that the addition of KH570 promoted the simultaneous formation of both low- and high-molecular-weight polymer species during polymerization, resulting in a highly heterogeneous molecular weight distribution (Ahmadi-Dehnoei et al., 2024). Such a broad molecular weight distribution may adversely affect the performance of the resulting sizing agent.

**TABLE 7:** Molecular weight distribution of Si_570_-F3.

Sample	M_w_ (g/mol)	M_n_ (g/mol)	PDI
0.5%Si_570_-F3	231,342	23,579	9.80
1% Si_570_-F3	453,069	25,033	18.1
1.5%Si_570_-F3	308,339	14,302	21.5
F3	21,444	6382	3.36

#### 3.3.4 Effect of KH570 content on the thermal stability of F3 sizing agent

Figure 11 shows the effect of KH570 on the thermal stability of the fluorinated acrylate sizing agent. The major weight loss occurred between 306 °C and 405 °C, corresponding to the thermal decomposition of the polymer chains. Further weight loss in the range of 405 °C–450 °C was mainly associated with the cleavage of C–C, C–O, and C–H bonds, after which the degradation rate gradually decreased. Above 450 °C, only a small amount of residual char remained. Although the initial decomposition temperature of the KH570-modified sizing agent was higher than that of F3, it remained lower than that of the KH560-modified sample. This difference is attributed to the epoxy groups of KH560, which participate in the crosslinking reaction and promote stronger interchain interactions, thereby resulting in better thermal stability (Liu et al., 2021).

**FIGURE 11:**
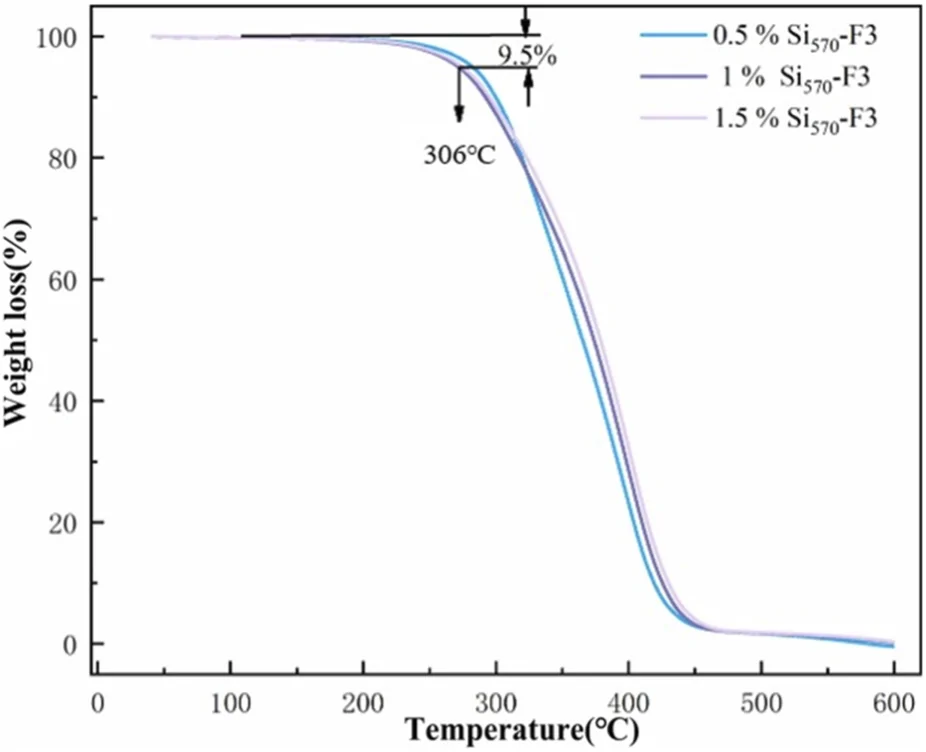
Thermogravimetric curves of Si_570_-F3 adhesive film.

#### 3.3.5 Effect of KH570 content on the water absorption of F3 sizing agent

To further investigate the effect of KH570 content on the water absorption of the sizing agent, the 24 h water absorption of the polymer films was measured. As shown in Figure 12, the water absorption gradually decreased with increasing KH570 content, from approximately 5% to 1%. Compared with the fluorinated sizing agent F3, the KH570-modified samples exhibited lower water absorption. Moreover, the water absorption of the sample containing 5 wt% KH570 was comparable to that of 5 wt% Si_560_-F3, with both samples showing similarly low water absorption.

**FIGURE 12:**
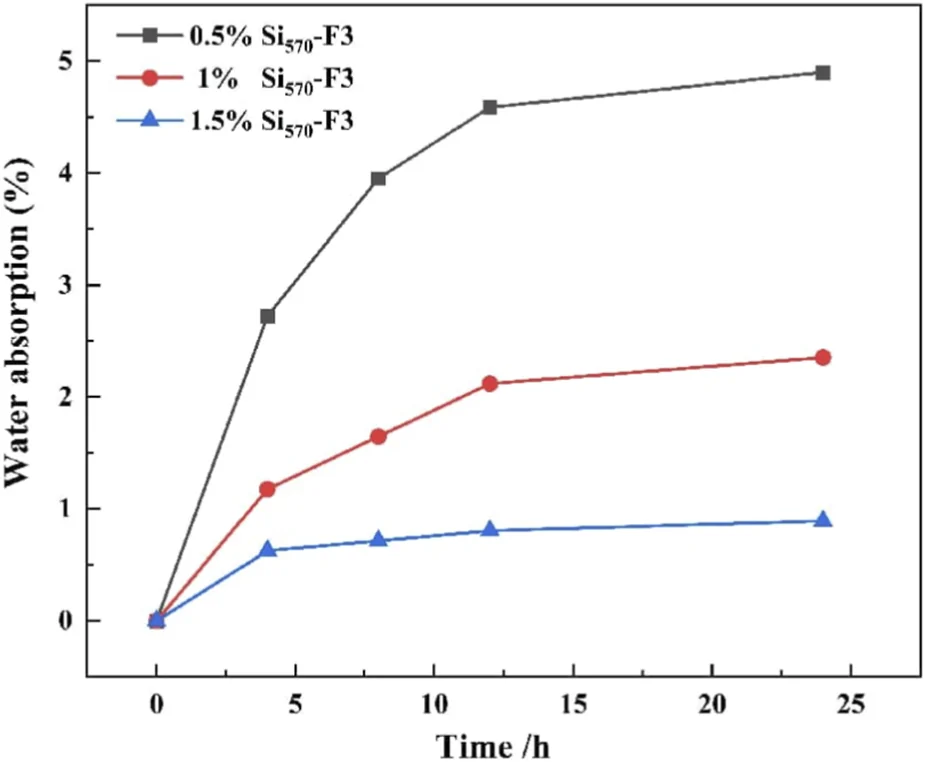
Effect of KH570 on the water absorption of adhesive film.

### 3.4 Properties and reinforcement mechanism of surface-sized specialty paper

#### 3.4.1 Mechanical properties testing of specialty paper-based materials

The mechanical properties of paper samples were evaluated before and after sizing treatment, with results compiled in Figure 13.

**FIGURE 13:**
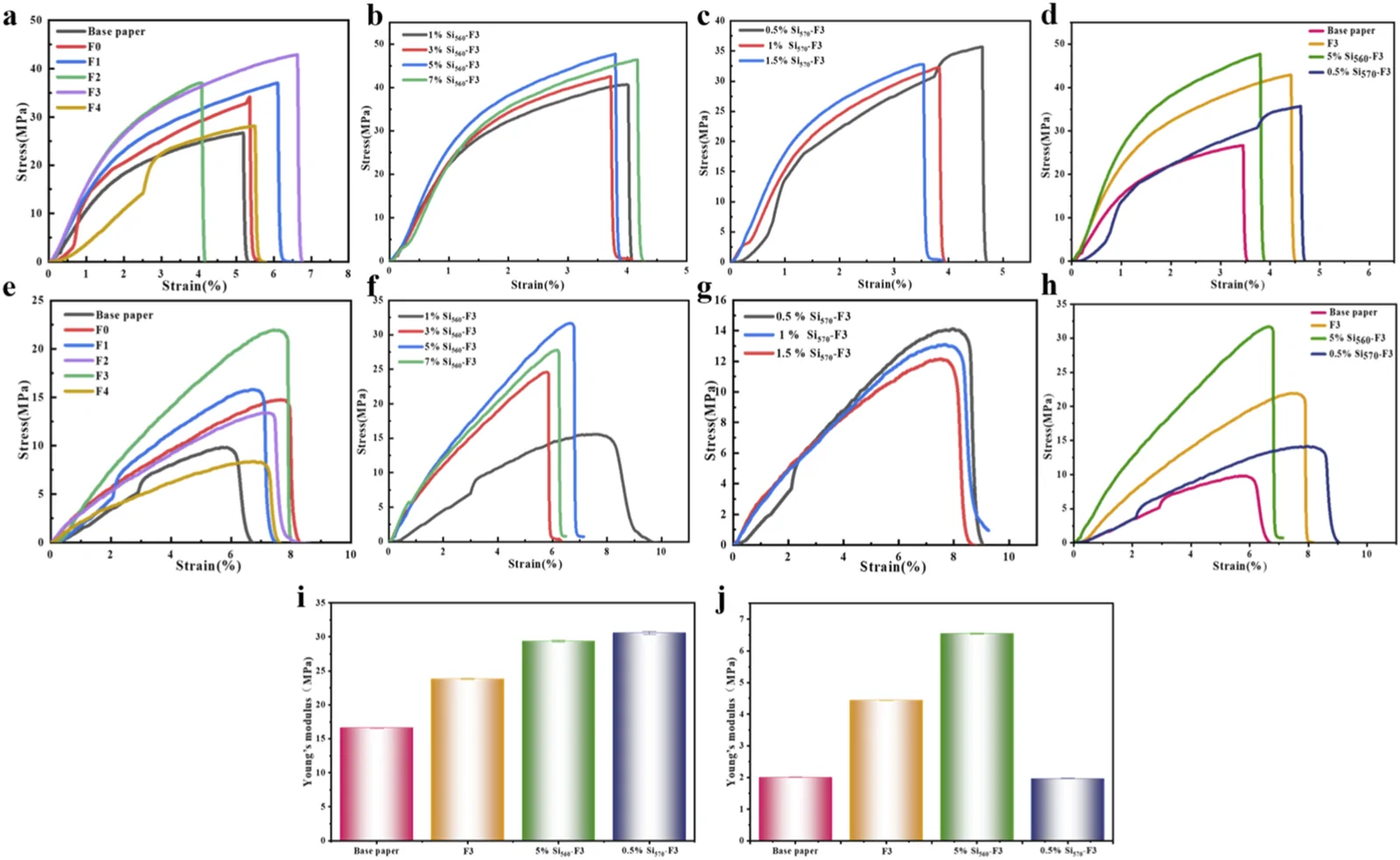
Tensile performance of sized paper: **(a)** dry tensile curves of the Fn series; **(b)** dry tensile curves of the Si_560_-F3 series; **(c)** dry tensile curves of the Si_570_-F3 series; **(d)** representative stress–strain curves of the untreated paper, F3, 5% Si_560_-F3, and 0.5% Si_570_-F3 under dry conditions; **(e)** wet tensile curves of the Fn series; **(f)** wet tensile curves of the Si_560_-F3 series; **(g)** wet tensile curves of the Si_570_-F3 series; **(h)** representative stress–strain curves of the untreated paper, F3, 5% Si_560_-F3, and 0.5% Si_570_-F3 under wet conditions; **(i)** Young’s modulus of the representative samples under dry conditions; and **(j)** Young’s modulus of the representative samples under wet conditions.

In the dry state (Figures 13a-c), the base paper exhibited an ultimate tensile strength (UTS) of 28.1 MPa. After treatment with the fluorine-modified acrylate sizing agent, the UTS increased to 42.9 MPa for F3, indicating that fluorinated acrylate sizing effectively enhanced the load-bearing capacity of the fiber network. Further modification with KH560 increased the UTS to 47.8 MPa for 5% Si_560_-F3, whereas 0.5% Si_570_-F3 showed a lower UTS of 35.7 MPa. This improvement can be attributed to the penetration of the sizing agent into interfiber voids and the formation of polymer bridges that enhance stress transfer within the fiber network (Gao et al., 2024). The representative stress–strain curves showed an initial approximately linear region followed by nonlinear deformation before failure (Figure 13d). The apparent Young’s modulus increased from 16.59 MPa for the base paper to 23.81 MPa for F3, 29.38 MPa for 5% Si_560_-F3, and 30.56 MPa for 0.5% Si_570_-F3, as summarized in Table 8. These results indicate that both fluorination and silane modification increased the initial stiffness of the paper network. However, the relationship between stiffness and ultimate strength differed for the two silane-modified systems. The 5% Si_560_-F3 sample combined a high modulus of 29.38 MPa with the highest UTS of 47.8 MPa, indicating improved stiffness and load-bearing capacity. In contrast, although 0.5% Si_570_-F3 exhibited the highest dry-state modulus of 30.56 MPa, its UTS was only 35.7 MPa. This suggests that a higher initial stiffness does not necessarily result in higher ultimate tensile strength. KH560 may provide more effective interfacial bonding through epoxy-related interactions and restrict fiber slippage, thereby maintaining stress transfer at larger deformation (Dai et al., 2024).

**TABLE 8:** Young’s modulus and ultimate tensile strength of representative paper samples under dry and wet conditions.

Sample	Dry E (MPa)	Wet E (MPa)	Dry UTS (MPa)	Wet UTS (MPa)
Base paper	16.59	2.00	28.1	8.30
F3	23.81	4.44	42.9	20.9
5% Si_560_-F3	29.38	6.54	47.8	32.8
0.5% Si_570_-F3	30.56	1.97	35.7	14.1

After 24 h of water immersion, both E and UTS decreased markedly for all samples (Figures 13e-j). The wet-state Young’s moduli of the base paper, F3, 5% Si_560_-F3, and 0.5% Si_570_-F3 were 2.00, 4.44, 6.54, and 1.97 MPa, respectively, while the corresponding wet UTS values were 8.30, 20.9, 32.8, and 14.1 MPa (Table 8). Among the four samples, 5% Si_560_-F3 retained the highest wet-state modulus and UTS, indicating better resistance to water-induced weakening. Its modulus retention was approximately 22.3%, compared with only about 6.4% for 0.5% Si_570_-F3. This difference suggests that the KH560-modified interfacial network is more effective in maintaining fiber–fiber stress transfer under wet conditions. The improved wet-state stability may arise from stronger hydrogen-bonding and epoxy-related interactions at the polymer–fiber interface (Wang et al., 2024d). Combined with the cross-sectional SEM observations, these mechanical results suggest that the reinforcement mainly originates from modification of the fiber surfaces and fiber–fiber interfaces rather than changes in the intrinsic mechanical properties of the cellulose fibers. The sizing agent fills interfiber voids and forms polymer-rich bridges between adjacent fibers, thereby improving interfacial load transfer and restricting fiber slippage.

#### 3.4.2 Water contact angle measurement of specialty paper

Water contact angles were measured for paper samples sized with Fn, Si_570_-F3, and Si_560_-F3 series, both before and after 24 h of water immersion (Figure 14). In all three systems, contact angles increased with higher loadings of HFBMA, KH570, or KH560—a direct consequence of the low surface energy imparted by the fluorinated segments and siloxane components, which reduce the surface polarity of the acrylate resin (Ma et al., 2024a). For the F3 system (Figure 14), the film contact angle topped at 109°. Sizing raised the dry paper contact angle from 86° (base paper) to 118°, which remained at 95° after immersion. In the Si_570_-F3 system (Figure 14), the maximum film contact angle was 101° at 1.5% loading; dry paper values went from 105° to 111°, then fell to 95.1° after wetting. The Si_560_-F3 system (Figure 14) gave a film contact angle of 107° at 5% loading; dry paper contact angles rose from 102° to 121°, and held at 98° after immersion. Interestingly, the sized paper consistently showed higher contact angles than the corresponding films. This owes to the paper’s intrinsic surface roughness, which works together with the low surface energy to enhance hydrophobicity (Wang et al., 2024c). The modest drop after water immersion likely stems from fiber swelling and increased surface polarity upon water uptake.

**FIGURE 14:**
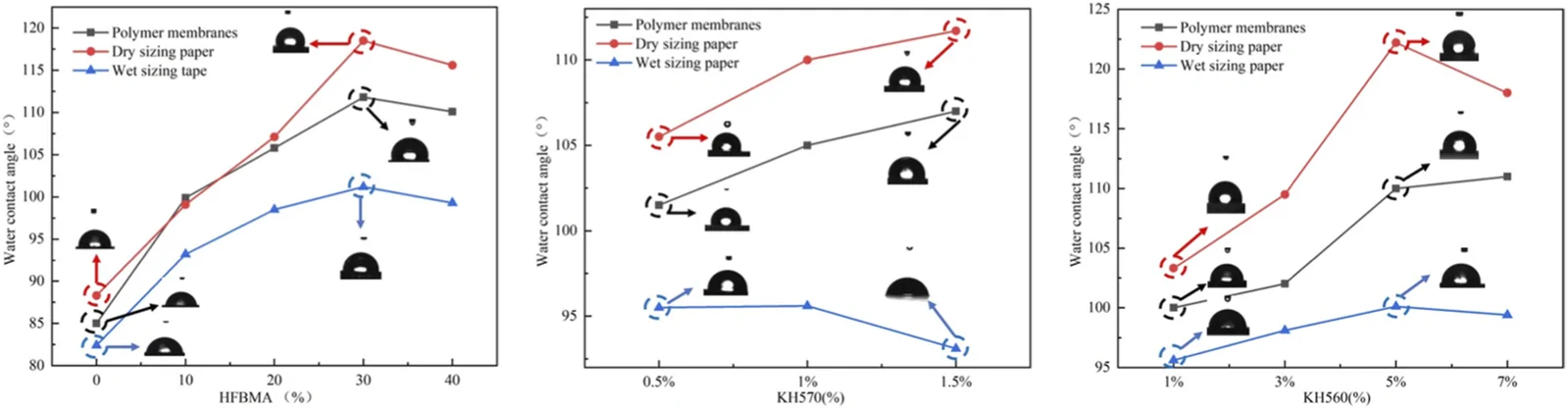
Test of water contact angle between adhesive film and sizing paper.

The water resistance obtained in this study was also compared with previously reported fluorinated or silicon-containing coatings. Zheng et al. reported a polycationic polysilazane coating on cotton fabrics with water contact angles above 134° (Zheng et al., 2026), which is higher than the approximately 122° obtained for the optimized Si_560_-F3-sized paper. This difference may mainly originate from the different coating architectures and substrates. The polysilazane system primarily forms a low-surface-energy layer on the textile surface, whereas the present sizing agent partially penetrates into the porous paper network while simultaneously forming a hydrophobic surface layer. Similarly, Song et al. prepared fluorinated polycationic coatings on textile substrates by initiated chemical vapor deposition, which favors the formation of a uniform fluorinated surface (Song et al., 2021). In the present system, HFBMA lowers the surface energy, while KH560 promotes interfacial bonding and network formation through epoxy ring-opening reactions and Si-O-Si structures. Therefore, although the water contact angle is somewhat lower than that of highly hydrophobic textile coatings, the optimized Si560-F3 treatment simultaneously increased the tensile strength from 28.1 to 47.8 MPa and reduced the 24 h water absorption from 29.1% to 5.5%, indicating a combined improvement in surface hydrophobicity, bulk water resistance, and mechanical reinforcement.

#### 3.4.3 Microstructural analysis of specialty paper

The morphology of paper samples before and after sizing was characterized using SEM. Unsized base paper contained abundant voids between loosely bonded fibers (Figure 15a). After sizing, polymer resin deposited on fiber surfaces and filled spaces between fibers. It formed adhesive bridges connecting neighboring fibers, which suggests some penetration into the porous paper matrix. Surface micrographs show relatively smooth coatings on Fn-sized paper (Figures 15b,c). Samples modified with KH560 or KH570 display fine granular protrusions across their surfaces (Figures 15d,e). These features may arise from local aggregation or crosslinking events during curing.

**FIGURE 15:**
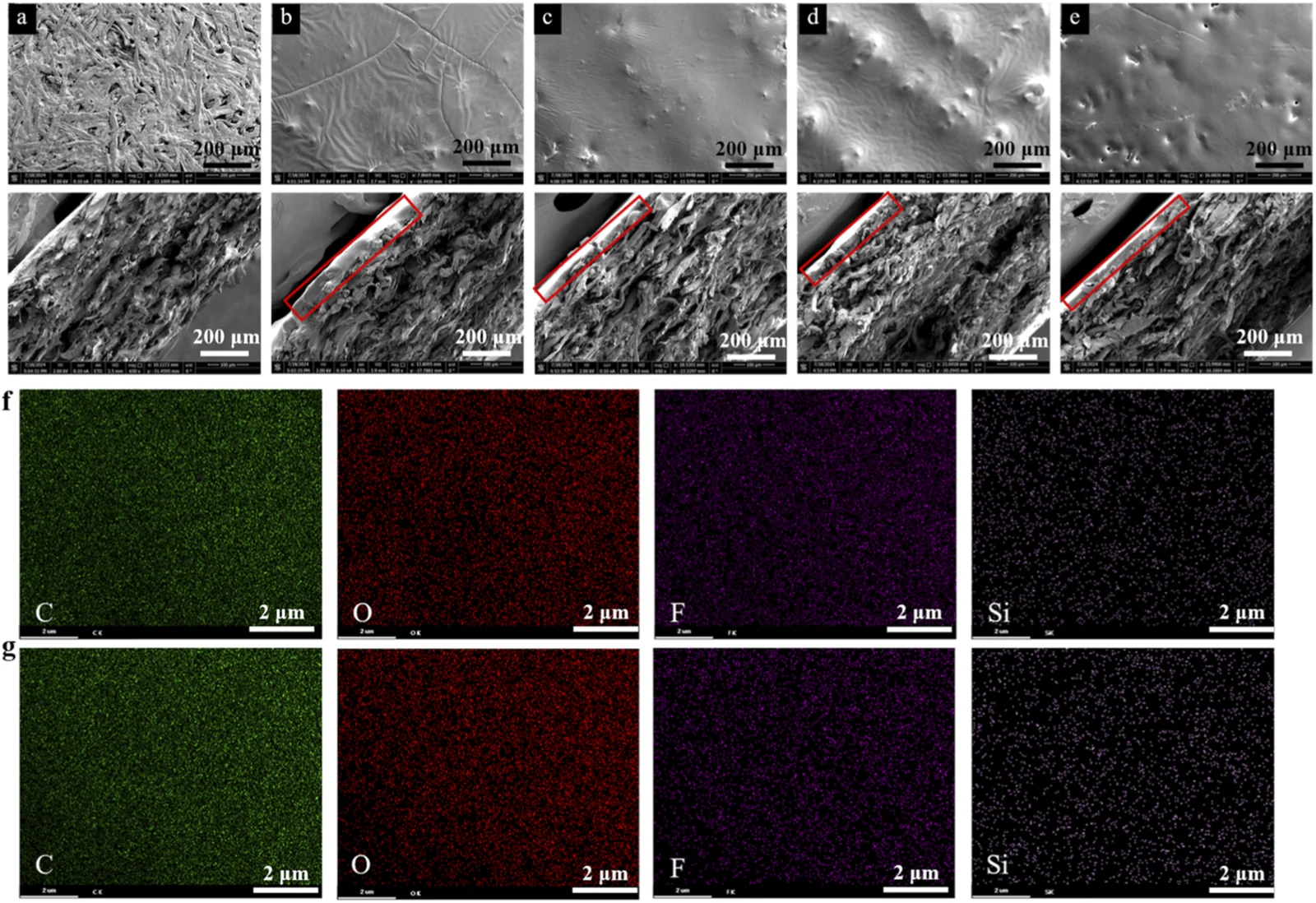
SEM diagram of paper plane and cross-section. **(a)** unglued paper; **(b)** F0 sizing paper; **(c)** F3 sizing paper; **(d)** 5%Si_560_-F3; **(e)** 0.5%Si_570_-F3; The following is a cross-sectional view of the corresponding paper; EDS test on paper: **(f)** The EDS image of the 5%Si_560_-F3 coated surface; **(g)** The EDS image of the 0.5%Si_570_-F3 coated surface.

Higher-magnification cross-sectional SEM of 5% Si_560_-F3 and 0.5% Si_570_-F3 samples identifies a fairly compact (Figure 16), continuous polymer-rich layer close to the paper surface. This layer adheres tightly to the upper fiber network. Sizing agent appears to accumulate preferentially near the paper surface while also migrating into gaps between fibers. Images cannot confirm uniform coating over every single fiber. Still, the dense near-surface region and visible interfacial bonding point to contributions from surface modification and reinforcement within the fiber network. Both effects likely improve paper performance. EDS elemental mapping (Figures 15f,g) reveals even distributions of C, O, F and Si across sized paper surfaces. The data supports successful incorporation of fluorine–silicon modified resin across the sample surface. Improved water resistance and wet tensile strength may therefore stem from combined surface hydrophobization and polymer reinforcement within the fiber network.

**FIGURE 16:**
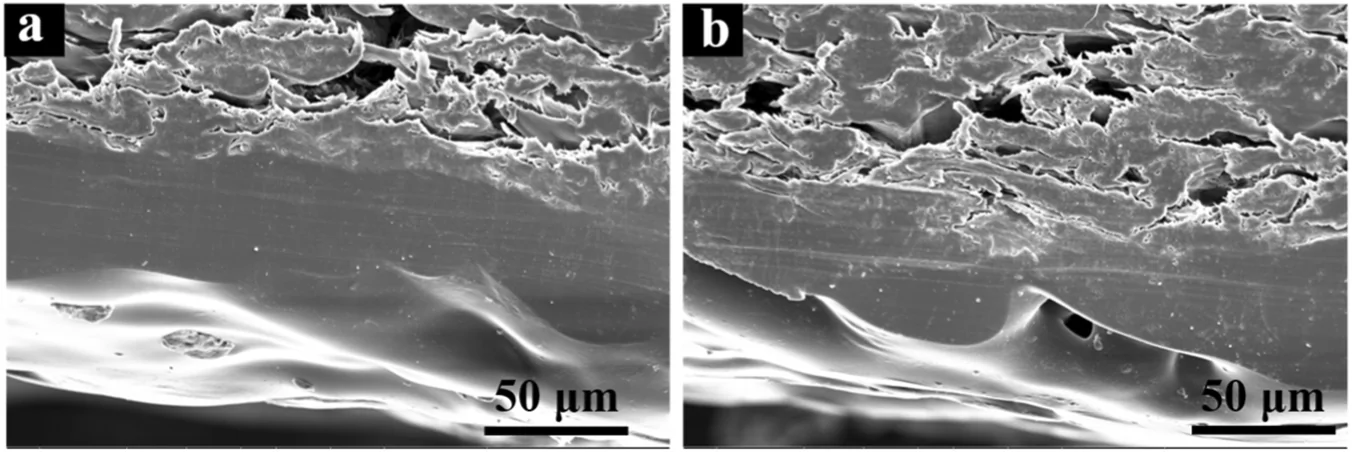
Enlarged cross-sectional SEM images of sized paper: **(a)** 5% Si_560_-F3; **(b)** 0.5% Si_570_-F3.

#### 3.4.4 Water absorption test of sized specialty paper

The sized paper samples prepared as described above were subjected to water absorption tests over 24 h, and the results are presented in Figure 17. All sized papers exhibited substantially lower water absorption than the unsized base paper. With increasing HFBMA content, the water absorption first decreased and then slightly increased, which is consistent with the hydrophobic nature of the fluorinated monomer (Figure 17a). For the KH570-modified series (Figure 17b), water absorption declined steadily from 29.1% (base paper) to a minimum of 6.90% as KH570 loading increased. In contrast, the KH560-modified papers showed a distinct minimum at 5% addition (5.50%), with water absorption decreasing initially and then rising slightly at higher KH560 levels (Figure 17c). This trend is attributed to the cooperative effect between KH560 and the C–F segments of HFBMA, which together form a moderately crosslinked Si–O–Si network on the paper surface that effectively impedes water penetration (Wang et al., 2024a). However, excessive KH560 introduces more organic coupling groups and crosslinking sites, leading to enhanced hydrogen bonding among polymer chains. This promotes molecular aggregation, which hinders uniform penetration into the fiber matrix and results in a modest increase in water absorption (Bang et al., 2024).

**FIGURE 17:**
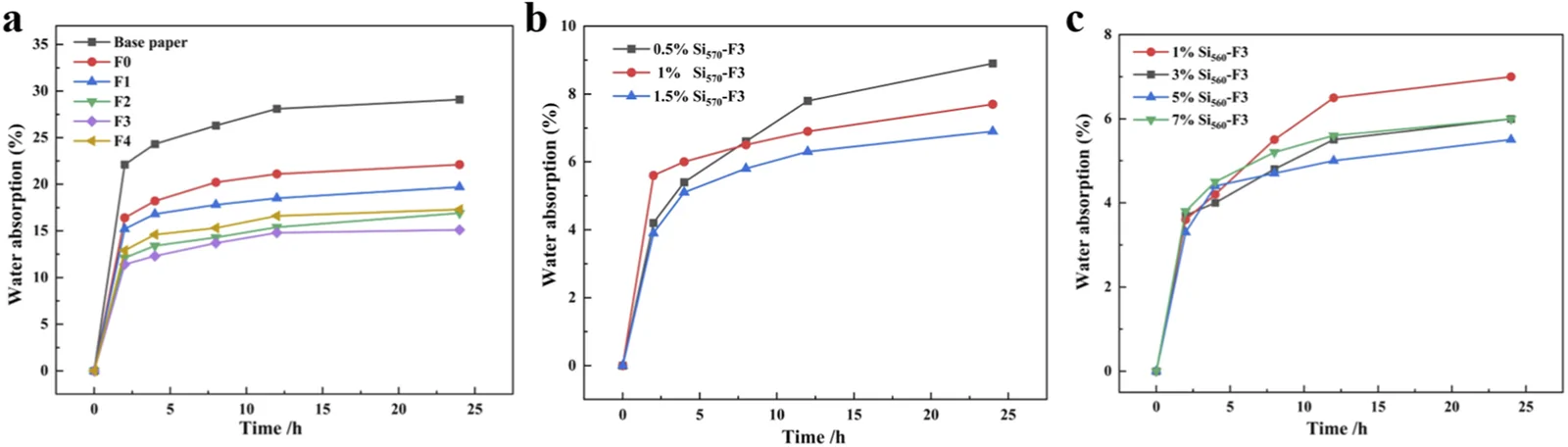
Water absorption test of sizing paper: **(a)** Water absorption curve of Fn series sizing paper; **(b)** Si_560_-F3 series sizing paper water absorption line; **(c)** Water absorption curve of Si_570_-F3 series sizing paper.

### 3.5 Mechanisms of water resistance and reinforcement for acrylate sizing agent

The proposed mechanism for water resistance and reinforcement of the sizing agent is illustrated in Figure 18. The polymer chains contain abundant hydroxyl, epoxy, and other reactive groups, which impart strong adhesion and adsorption to cellulose fibers through hydrogen bonding and other intermolecular interactions. The introduction of HFBMA brings C–F bonds with higher bond energy than other bonds in the polymer backbone, which improves thermal stability, chemical stability, and contributes to the extremely low surface energy of the system (Hukum et al., 2024). The silane coupling agents further introduce Si–O–Si crosslinking sites and reactive–Si(OH)_3_ groups, enhancing hydrogen bonding both among polymer chains and between the polymer and cellulose fibers. After curing, the fluorinated segments migrate to the paper surface and arrange themselves into a low-surface-energy hydrophobic layer. The siloxane components not only reduce surface energy but also strengthen intermolecular forces, while the increased crosslink density provides higher mechanical strength to the cured film. The sizing agent also penetrates into the voids between fibers, obstructing water molecules from passing through and thereby improving water resistance. In addition, hydrogen bonds, covalent crosslinks, and intermolecular forces work together to form a three-dimensional network that further enhances the overall mechanical properties of the specialty paper (Deng et al., 2023).

**FIGURE 18:**
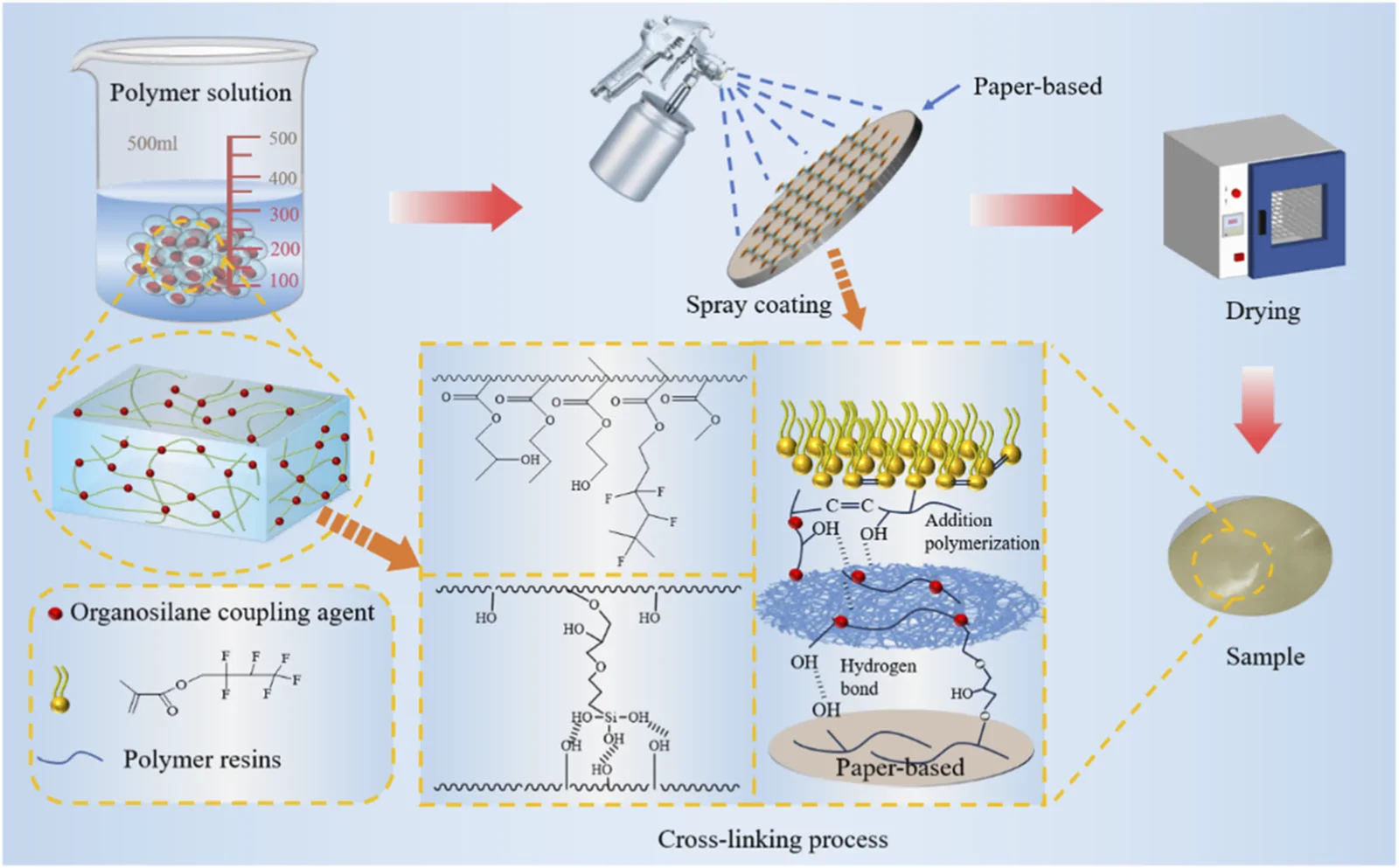
Reinforcement mechanism of sizing agent on paper.

## 4 Conclusion

A series of fluorine-modified acrylate resins (Fn) were synthesized by radical polymerization. Two fluorine–silicon co-modified sizing agents, Si_570_-F3 and Si_560_-F3, were further prepared via different reaction routes using hexafluorobutyl methacrylate (HFBMA) as the fluorinated monomer, together with MMA, BA, HPA, and HEMA. Adding HFBMA clearly improved both thermal stability and sizing performance. Between the two silane coupling agents, KH560 outperformed KH570 in enhancing thermal stability. Mechanical and water-resistance tests on the sized specialty papers gave consistent results. The F3-treated paper reached a tensile strength of 42.9 MPa (up 52.7% over the base paper), with a drop in water absorption from 29.1% to 15.1% (a 48.1% reduction). The 0.5% Si_570_-F3 formulation yielded a tensile strength of 35.7 MPa (+27.0%) and cut water absorption by 73.5%. Best overall performance came from 5% Si_560_-F3, which delivered a tensile strength of 47.8 MPa (+70.1%), an elongation at break of 3.80%, a water absorption reduction of 81.1%, and an increase in water contact angle from 85° to 122°. In conclusion, these findings show that the solvent-based fluorine–silicon modified acrylate sizing agent offers a practical route to significantly boost both the mechanical strength and water resistance of specialty paper, making it attractive for high-performance sizing applications.

## Data Availability

The original contributions presented in the study are included in the article/supplementary material, further inquiries can be directed to the corresponding authors.
